# Development of a New Highly Selective Monoclonal Antibody against Preferentially Expressed Antigen in Melanoma (PRAME) and Identification of the Target Epitope by Bio-Layer Interferometry

**DOI:** 10.3390/ijms22063166

**Published:** 2021-03-20

**Authors:** Jwala Priyadarsini Sivaccumar, Antonio Leonardi, Emanuela Iaccarino, Giusy Corvino, Luca Sanguigno, Angela Chambery, Rosita Russo, Mariangela Valletta, Debora Latino, Domenica Capasso, Nunzianna Doti, Menotti Ruvo, Annamaria Sandomenico

**Affiliations:** 1 Istituto di Biostrutture e Bioimmagini, CNR, 80134 Napoli, Italy; jwala.priyadarsini@gmail.com (J.P.S.); emanuela.iaccarino@gmail.com (E.I.); giusycorvino1986@gmail.com (G.C.); latinodebora@gmail.com (D.L.); nunzianna.doti@cnr.it (N.D.); 2 Dipartimento di Medicina Molecolare e Biotecnologie Mediche, Università degli Studi di Napoli Federico II, 80142 Napoli, Italy; leonardi@unina.it (A.L.); lucasanguigno@libero.it (L.S.); 3 Dipartimento di Scienze e Tecnologie Ambientali, Biologiche e Farmaceutiche (DISTABIF), Università L. Vanvitelli, 80100 Caserta, Italy; angela.chambery@unicampania.it (A.C.); rosita.russo@unicampania.it (R.R.); mariangela.valletta@unicampania.it (M.V.); 4 Centro di Servizio di Ateneo per le Scienze e Tecnologie per la Vita (CESTEV), Università di Napoli Federico II, 80145 Napoli, Italy; domenica.capasso@unina.it

**Keywords:** PRAME, mAb, bio-layer interferometry, epitope identification

## Abstract

Background: Monoclonal antibodies (mAbs) against cancer biomarkers are key reagents in diagnosis and therapy. One such relevant biomarker is a preferentially expressed antigen in melanoma (PRAME) that is selectively expressed in many tumors. Knowing mAb’s epitope is of utmost importance for understanding the potential activity and therapeutic prospective of the reagents. Methods: We generated a mAb against PRAME immunizing mice with PRAME fragment 161–415; the affinity of the antibody for the protein was evaluated by ELISA and SPR, and its ability to detect the protein in cells was probed by cytofluorimetry and Western blotting experiments. The antibody epitope was identified immobilizing the mAb on bio-layer interferometry (BLI) sensor chip, capturing protein fragments obtained following trypsin digestion and performing mass spectrometry analyses. Results: A mAb against PRAME with an affinity of 35 pM was obtained and characterized. Its epitope on PRAME was localized on residues 202–212, taking advantage of the low volumes and lack of fluidics underlying the BLI settings. Conclusions: The new anti-PRAME mAb recognizes the folded protein on the surface of cell membranes suggesting that the antibody’s epitope is well exposed. BLI sensor chips can be used to identify antibody epitopes.

## 1. Introduction

Cancer is a disease with great molecular diversity and unpredictable nature. To combat its complexity and achieve improved treatment outcomes, modern oncology is shifting from empirical treatment strategies to biomarker-driven treatment models based upon the molecular profile of tumors from single individuals. The development of personalized cancer therapy is reliant on the identification and validation of specific biomarkers, which are associated, or even coincident, with the therapeutic targets whose activity is modulated by the administered drugs. In cancer, most drugs are monoclonal antibodies able to bind with high affinity and selectivity specific sites of the targets, thus preventing pathologically relevant interactions or blocking aberrant activities [1]. In this context, knowing the antibody’s epitope on the target protein is of utmost importance to plan the antibody use and to understand its therapeutic potential. One group of tumor-specific biomarkers called preferentially expressed antigen of melanoma (PRAME), also known as MAPE (melanoma antigen preferentially expressed in tumors), cancer-testis antigen 130 (CT130), and OIP4 (OPA-interacting protein 4) was initially identified in an autologous cytolytic T lymphocyte clone in a melanoma cell line [2]. Although PRAME belongs to the category of cancer-testis antigen, it is aberrantly reexpressed in many types of cancers, including metastatic melanoma, head and neck carcinoma, renal cell cancer, multiple myeloma, non-small cell lung carcinomas, neuroblastoma, chronic myeloid leukemia, acute leukemia, uveal melanoma, several sarcoma subtypes, and in triple-negative breast cancers. In most of them, its presence is associated with a poor prognosis [3]. High levels of PRAME expression are correlated with favorable outcomes following chemotherapy treatments of hematological malignancies, such as acute myeloid and lymphoblastic leukemia [4,5].

PRAME is a member of the leucine-rich repeat (LRR) family of proteins and physiologically acts mainly by inhibiting the retinoic acid-mediated differentiation, proliferation and apoptosis [6]. However, the precise molecular functions of PRAME and its role in oncogenesis are not well understood. Hence, far, by epitope-tagged immunoprecipitations and mass spectrometry, it has been established that PRAME facilitates the recruitment of cullin2 ubiquitin ligases to the EKC/KEOPS complex in the nucleus where it is involved in the transcriptional regulation of target genes [7,8,9]. Several studies have also shown that upregulation of PRAME expression in various types of malignancies is linked to hypomethylation of DNA promoters [10,11,12,13,14]. Furthermore, the upregulation of PRAME features cell stemness, invasion, and metastasis in triple-negative breast cancer by promoting the epithelial-to-mesenchymal transition (EMT) through the activation of ZEB1 and downregulation of *BMP7* and *TSPAN13* genes) [15]. By all these features, PRAME is emerging as an interesting biomarker and a potential therapeutic target for a number of diseases [3]. Several immunotherapeutic clinical trials targeted PRAME by means of PRAME peptides and adoptive T cell therapy with autologous pre-existing circulating PRAME-specific T cells or genetic engineering of high-affinity PRAME-specific TCR T cells [3,16].

Numerous evidence also indicates that PRAME is membrane-bound in several cancer cells and that antibodies targeting the extracellular region 310–331 show effectiveness to detect cancers and potentially treat them through targeted therapies [17]. On the other hand, a TCR mimicking a human antibody was developed to recognize a PRAME peptide (300–309) in complex with HLA-A2, enabling antibody-dependent cellular cytotoxicity (ADCC) [18]. Further, anti-PRAME TCR mimetics can enhance antibody-dependent phagocytosis on PRAME-positive cancer cells by synergistic treatments with CD47 blockade agents [19].

All these recent evidences support PRAME as a promising target for developing CAR-T-based immunotherapies using CARs that bind PRAME peptides bound to HLAs or the protein on cancer cells expressing it on their surface. They also support the use of antibodies and related drug conjugates, alone or in combination with immune checkpoint inhibitors, for the targeted therapy of PRAME-positive cancers.

The epitopes recognized by antibodies obtained by immunization with full-length folded proteins are often unknown and may request extensive investigations with overlapping synthetic peptides or mutational studies. Antibodies raised against small synthetic peptides have the advantage that the binding site is known a priori. However, they may display a relatively low affinity for the full-length parent proteins where the structure of the epitope may differ considerably from that of the original immunogen. In the case of PRAME, the epitope analysis of a panel of mAbs [20] and the development of a polyclonal antibody against the predicted extracellular PRAME 310–331 peptide [16] have been recently described. Capture experiments of protein fragments with antibody-immobilized sensor chips can also be performed in this instance using, for example, label-free devices that provide real-time evidence of the capture and of the release of the bound epitope [21,22]. Instruments working in flow are well suited for these purposes; however, dilution of the sample and difficulties in collecting the bound fractions may represent an important limitation.

Here, we report the generation of a monoclonal antibody against the central protein domain of PRAME spanning residues 161 to 415. The selected murine monoclonal antibody, named 2D5, exhibits a very strong affinity for the protein (dissociation constant in the picomolar range), binds and efficiently recognizes the native PRAME protein in Western blotting and FACS analyses. The epitope of the antibody also has been identified by immunoaffinity capturing the trypsin digested protein fragments on a bio-layer interferometry sensor chip derivatized with the antibody, taking advantage of the small volume used in the experiments and the lack of fluidics.

## 2. Results

### 2.1. Proteins Expression and Purification

The human PRAME protein (UniProtKB—P78395) spanning residues 161–415 was expressed in *E. coli* as N-terminal His-tagged recombinant protein using the pET28a vector (*rh*PRAME, Figure S1). The recombinant protein was mostly recovered after repeated dialysis against PBS containing 0.5 M urea and 1 mM DTT with a purity higher than 90%, as demonstrated by SDS–PAGE analysis on 12% polyacrylamide gel. The protein was detected as a band at MW between 37 kDa and 25 kDa in agreement with the predicted molecular weight of about 33 kDa (Figure S2A,B). Lower concentrations of urea lead to precipitation of most protein; therefore, the final sample was stored in a final buffer containing the denaturing agent at 0.5 M. However, some diluted protein samples were recovered and characterized by gel filtration to assess that it was not aggregated and used to perform immunization of the animals for producing the monoclonal antibodies, the ELISA and the SPR assays. GF analysis of the pure protein showed that the fraction recovered after urea removal was monomeric and highly pure (See Figure S3A–C). The anti–PRAME 2D5 mAb was obtained >90% pure as assessed by 15% SDS–PAGE under reducing and nonreducing conditions (Figure S4).

### 2.2. Binding of 2D5 Anti-PRAME mAb to rhPRAME by ELISA and SPR

**Figure 1:**
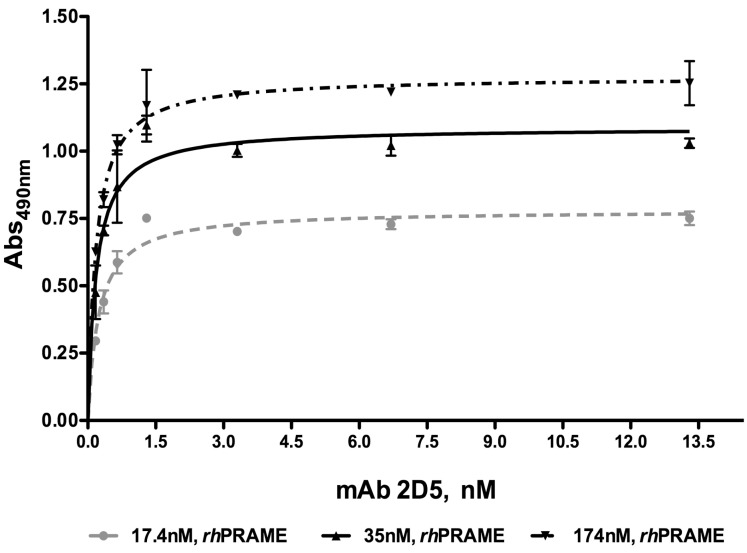
ELISA testing of the binding between the anti-preferentially expressed antigen in melanoma (PRAME) 2D5 mAb and *rh*PRAME) coated at the reported concentrations. A K_D_ of 0.19 ± 0.03 nM was roughly estimated as the average value of the least 3 independent experiments.

**Figure 2:**
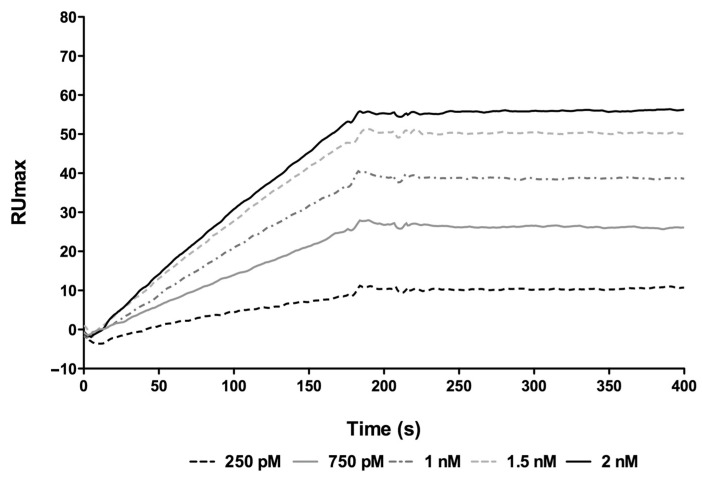
SPR dose–response assays between the immobilized *rh*PRAME and the anti-PRAME 2D5 mAb at various concentrations. The overlaid sensorgrams obtained at mAb concentrations between 250 pM and 2.0 nM are shown. All experiments were carried out at 25 °C at a constant flow rate of 20 μL/min using HBS-EP as a running buffer. Kinetic parameters are reported in Table 1.

The selected and purified murine anti-PRAME 2D5 monoclonal antibody was tested for its capability to properly recognize the *rh*PRAME by ELISA (Enzyme linked Immunosorbet assay) and SPR ( Surface Plasmon Resonance) direct binding assays. As shown in Figure 1, the 2D5 mAb recognized the coated *rh*PRAME protein in a dose-dependent and saturable manner. A rough estimation of the affinity obtained by fitting the data points with a non linear algorithm provided a K_D_ of 198 ± 34 pM. We also performed a preliminary comparison of the binding to *rh*PRAME between the new mAb and a commercially available polyclonal antibody (Abcam code ab89097). Data preliminarily showed that 2D5 bound to a greater extent the protein compared to the commercial product (Figure S5), although it must be noted that the commercial product was a polyclonal antibody, thus likely at a lower effective concentration generated against the full-length protein. To further characterize this interaction, we performed dose-dependent binding assays through SPR immobilizing the protein on a CM5 sensor chip. As shown in Figure 2, the 2D5 mAb bound very efficiently and dose-dependently the immobilized *rh*PRAME, exhibiting an apparent affinity constant (K_D_) of 34.9 ± 5.0 pM. The strong affinity is mostly derived from the very slow dissociation rate (k_d_ = 4.51 × 10^−5^ s^−1^). Binding to the immobilized protein was already nearly saturated using the antibody at 2.0 nM. Association and dissociation rate constants and related kinetics parameters for all binding experiments are reported in Table 1.

### 2.3. Detection of Endogenous PRAME in Cell-Based Assays

**Figure 3:**
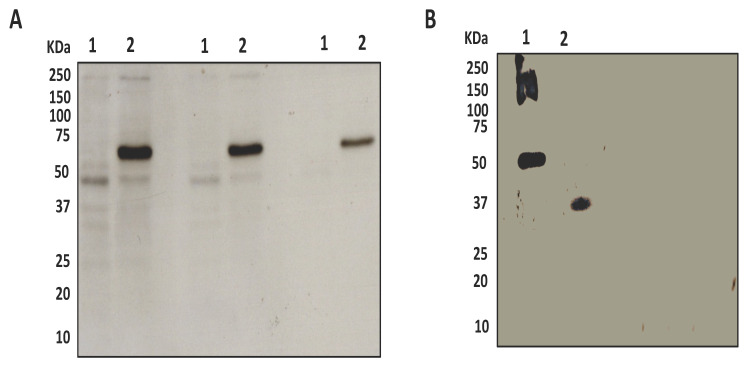
(**A**) Western blotting analysis of endogenous PRAME in L1236 (PRAME-positive) and KG1 (PRAME-negative) cell extracts using the mAb 2D5 at various concentrations. (**B**) Western blotting analysis of U2OS cell extracts using the mAb 2D5 at 5.0 µg/mL. In (**A**) M: markers; 1: KG1 cell extract probed with 2D5 at 5.0 µg/mL; 2: L1236 cell extract probed with 2D5 at 5.0 µg/mL; 3: KG1 cell extract probed with 2D5 at 2.0 µg/mL. 4: L1236 cell extract probed with 2D5 at 2.0 µg/mL; 5: KG1 cell extract probed with 2D5 at 1.0 µg/mL; 6: L1236 cell extract probed with 2D5 at 1.0 µg/mL. In (**B**) M: markers; 1: U20S (PRAME-positive) cell extract; 2: *rh*PRAME used as a positive control.

**Figure 4:**
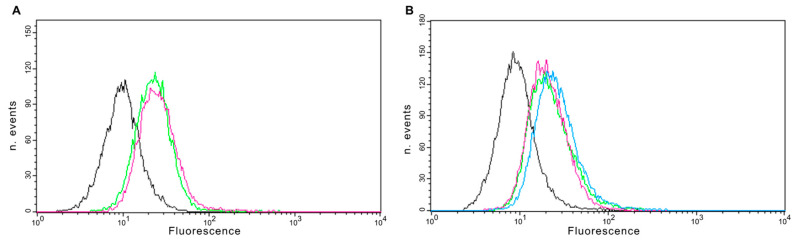
Flow cytometry analysis of U2OS cells using the 2D5 monoclonal antibody and the commercial antibody code ab89097, used as a positive control. (**A**) Cells at a density of 5 × 10^7^ cells/mL were incubated with 15 µg/mL of the commercial anti-PRAME antibody (green curve) or 2D5 mAb (magenta curve). (**B**) Cells were incubated with 7.5 (magenta curve), 15 (green curve) or 30 µg/mL (blue curve) of anti-PRAME 2D5 mAb. Black curves represent the cells incubated with isotype control antibody. These pictures are representative of different independent experiments.

The binding specificity of the anti-PRAME 2D5 mAb was assessed by its ability to detect the endogenous PRAME by Western blotting and FACS analysis in cancer cell lines expressing the protein. For this purpose, we used the L1236 and U2OS cancer cells, whereas the KG1 cell line, not expressing the protein, was selected as a negative control. As shown in Figure 3A,B, the 2D5 mAb was able to detect the endogenous PRAME protein by WB in both the L1236 (Figure 3A)- and U2OS (Figure 3B)-positive cell lines, while, as expected, no bands were detected in the PRAME-negative KG1 cells. We next performed a flow cytometry analysis on U2OS cells by combining cell surface and intracellular marker labeling. A commercial anti-PRAME antibody (Abcam, ab89097) and an IgG1 (isotype control) were used as positive and negative controls, respectively. The U2OS cells were harvested and incubated with either the commercial anti-PRAME antibody or the 2D5 mAb; following this step; cells were permeabilized and incubated again with the primary antibodies. As shown in Figure 4A, the cells treated with the 2D5 mAb displayed a significant shift of fluorescence intensity as compared to cells incubated with the isotype control. The same increase of fluorescence intensity was observed when the cells were treated with the commercial anti-PRAME antibody used at the same concentration. The data (Figure 4B) show that the binding signal was saturated at the lowest antibody concentrations (7.5 μg/mL).

### 2.4. Identification of the mAb 2D5 Epitope on rhPRAME

**Figure 5:**
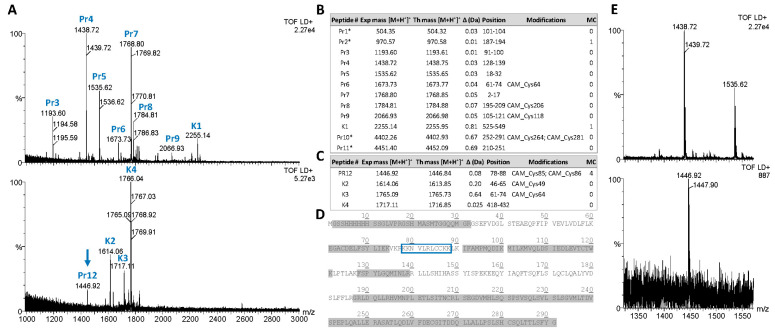
(**A**) Matrix-assisted laser desorption ionization time-of-flight (MALDI-TOF) spectra in the *m/z* range 1000–3000 of tryptic peptides (upper panel) and antibody-bound eluted peptides (lower panel) of human recombinant PRAME protein. (**B**) List of tryptic peptides from human recombinant PRAME protein (Pr) and human keratins (K) as identified by MALDI-TOF MS analyses on the digested protein. (**C**) List of tryptic peptides from the antibody-bound fraction eluted from the sensor tip as identified by MALDI-TOF MS analysis. Peptide’s theoretical and experimental masses, along with mass errors, amino acid positions and modification and the number of trypsin missed-cleavage sites, are reported (*, peptide ions not shown in MALDI-TOF spectra). (**D**) Tryptic peptides identified by MALDI-TOF MS analysis and mapping on the PRAME protein region 161–415. Sequence regions corresponding to the mapped tryptic peptides are shaded gray. The His-tag-containing linker (His-Tag) at the protein N-terminus is bold and underlined. The protein region 202–212, corresponding to the peptide uniquely detected in the antibody-bound sample, is blue boxed. (**E**) Magnification of the *m/z* region containing the ion signal at *m/z* 1446.92, uniquely detected in the sample of antibody-bound peptides.

To identify the protein epitope recognized by the anti-PRAME 2D5 mAb, an aliquot of the antibody was immobilized on the surface of the bio-layer interferometry (BLI) sensor chip (ForteBio, Fremont, CA, USA). The binding to *rh*PRAME was first assessed in preliminary experiments (Figure S6), exposing the sensor chip to a 1.0 µM solution of the intact protein, the same concentration as the digested protein. Remarkably all experiments were performed in a cuvette of only 4 µL total volume; therefore, only small amounts of material were used in these assays. Next, the sensor chip was exposed to the mixture of peptides obtained by treating the protein with trypsin. No binding was observed in this case due to the inability of the system to detect analytes with a molecular weight below around 7 kDa [23]. The bound fraction was eluted using a solution at pH 2.0 (0.1% TFA in H_2_O, 4.0 µL) and collected in a plastic tube. The procedure was repeated 10 times, collecting the eluted fraction always in the same tube to accumulate the material for the mass spectrometry analysis. The final sample, concentrated under vacuum up to about 10 µL, was analyzed by MALDI-TOF mass spectrometry (MS). The starting mixture was analyzed in a parallel experiment, as described in the section of Methods. A peptide mapping of the protein by MALDI-TOF MS (Figure 5A, upper panel) showed a sequence coverage of 66%, which is rather extensive considering the non-optimal reducing and denaturing conditions (0.5 urea) used to retain the protein solubility during the digestion (Figure 5D). The MALDI-TOF analysis of the bound peptides (Figure 5A, lower panel and Figure 5C) showed that, besides several ion signals deriving from human keratin contaminants, a clear ion signal at *m/z* 1446.92 was uniquely detected in the sample. This signal corresponded to the monoisotopic ion of fragment 202–212 of PRAME (see arrow in Figure 5A, lower panel; (R)KKNVLRLCCKK(L), theoretical monoisotopic [M + H]^+^ mass 1446.84 Da, Δ = −0.08 Da). Worth of note, this peptide, containing four missed cleavage sites (see magnification in Figure 5E), was undetectable within the whole tryptic digest, a finding strongly suggestive of the highly selective enrichment deriving from the binding to the immobilized antibody.

### 2.5. ELISA and BLI Binding between Biotin-PRAME Peptides and the Anti-PRAME 2D5 mAb

**Figure 6:**
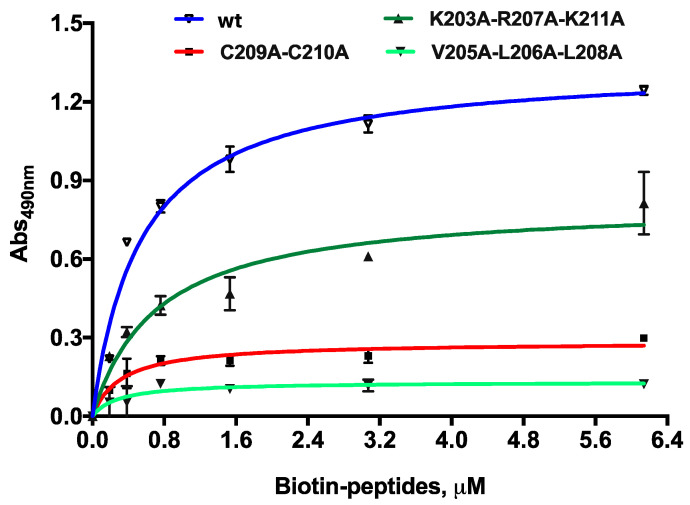
Dose–response ELISA binding of the biotin-PRAME (202–212) wild-type and mutanted peptides to the coated 2D5 mAb. For the wild type peptide, an affinity constant of 0.55 ± 0.10 μM was roughly estimated. The binding to the mutated variants under the same conditions are also reported.

The synthetic peptide reproducing the potential epitope, spanning residues 202–212 of *rh*PRAME (See Table S1), was next used in ELISA binding assays with the anti-PRAME 2D5 mAb to assess its recognition by the mAb. As shown in Figure 6, biotin-PRAME (202–212) bound in a dose-dependent manner the anti-PRAME 2D5 mAb, exhibiting an affinity constant estimated to be 0.55 ± 0.10 μM, much lower compared to that displayed by the protein, which is estimated to be between of 33.4 (SPR) and 198 pM (ELISA). Given the much lower affinity compared to the recombinant protein antigen, we investigated the specificity of the binding by designing and testing, in the same assay, a set of mutated peptide variants. Peptide sequences are reported in Table S1 together with that of the parent molecule. The 3 new peptides are (i) mutant K203A-R207A-K211A where the basic residue K203, R207 and K211 were replaced by alanine; (ii) mutant V205A-L206A-L208A where the hydrophobic residue V205, L206 and L208 were changed to alanine and (iii) mutant C209S-C210S where the 2 cysteines were replaced with the structurally homologous residue serine. As can be seen again in Figure 6, while mutants V205A-L206A-L208A and C209S-C210S completely lost the ability to bind to the antibody, the peptide K203A-R207A-K211A where the basic residues were replaced with alanines retained part of the affinity with 2D5. These observations were fully confirmed in parallel assays performed by BLI using the same peptides (See Figure S7A–D). Data indeed showed that the mutated peptide K203A-R207A-K211A was the only able to retain some affinity for the antibody and that the wt peptide exhibited a KD (0.59 µM) similar to that measured by ELISA (Figure S8). This observation suggests that most contribution to binding is provided by the hydrophobic residues and by cysteines, while a minor role is played by the positively charged lysines and arginines. A possible role in the recognition played by covalent bonds formed through the reactive thiols of the cysteines side-chains is ruled out by the observation that mutant V205A-L206A-L208A is fully unable to bind the antibody. The data thus indicate that the antibody recognition is specific for this protein region, although the reduced binding strongly indicates that other protein sites are involved and contribute to achieving an affinity in the pM range. The robust binding observed with the protein also opens the hypothesis that the real epitope might be conformational, thus strongly dependent on protein folding and structure, or it extends outside the N- or the C-terminus of the 202–212 fragment, including other residues removed by trypsin.

## 3. Discussion

Although hundreds of thousands of different antibodies are commercially available against essentially any protein (see, for example, www.proteinatlas.org/about/antibodies (accessed on 14 November 2020)), many others are continuously developed to fulfill the need of ever more selective and sensitive reagents for both the detection of biomarkers and the modulation of therapeutically relevant biological activities. One critical prerequisite for the success of an antibody is the knowledge of its binding site on the target protein, information that is often missing also for many commercial products. The binding site can be identified by various approaches, including site-directed mutagenesis, through the use of synthetic peptides or by fragmenting the protein and isolating the binding fragment by various approaches.

Recently, an epitope analysis of a panel of mAbs targeting PRAME protein has been reported [20]. Here, we explored the used of BLI biosensors to capture a fragment of the PRAME protein recognized by a newly generated monoclonal antibody.

Unlike many other CTAs, PRAME belongs to the leucine-rich repeat (LRR) family of proteins. Its localization on cell membranes [24] makes it a suitable diagnostic cancer biomarker and a target appropriate for developing diagnostic, monoclonal antibodies. The precise functions of PRAME and its contribution to oncogenesis are so far poorly understood; therefore, inhibitors can be difficult to design and develop.

Beyond its involvement in DNA hypomethylation [10,15], one leading hypothesis for its antiapoptotic activity relies on its ability to repress, in a cancer-specific manner, the expression of the potent tumor suppressor TRAIL through the binding to RAR (retinoic acid receptor) and to EZH2 that epigenetically represses TRAIL gene expression [25,26]. However, one such mechanism occurs within the cell, and the binding sites are unknown; thus, inhibitors like monoclonal antibodies cannot be easily managed. No mechanisms associated with its cell-surface localization have been so far reported, thus given its selective expression on several cancer cells, including lymphoma cells [4,5], the use of antibodies against the outer protein regions can be envisaged for CAR-T-based immunotherapies where no specific functions of the protein must be modulated. Recent evidence has indeed shed light on PRAME as a cell-surface cancer biomarker [17] and as a target for TCR mimic antibodies (TCRm) [18,19], opening the possibility for effective CAR-based T cell therapies. We found that our antibody binds to a region located on the protein second LRR domain. Basing on the ability of the 2D5 antibody to bind the protein in cytofluorimetry experiments and on the very hydrophilic nature of the composing residues, the region is likely well exposed on the outer protein surface. However, any further hypothesis is precluded by the lack of information on the protein structure. The huge difference in affinity exhibited by the isolated epitope compared to the full-length protein (around 550 nM compared to 35 pM, which is about 17,000-fold lower) also indicates that the antibody binds much better the epitope when it is embedded in the protein tridimensional structure where it likely adopts a more structured conformation. This observation strongly suggests the intriguing hypothesis that the antibody recognizes a specific protein conformation that is lost in the isolated peptide, thus leading to the highly reduced affinity. However, the ability of 2D5 to detect the protein in Western blotting suggests that the affinity for the unfolded protein is still sufficiently high for analytical applications and that also other protein residues contribute to the recognition. Given the properties exhibited by the antibody, including the high affinity (K_D_ = 33.4 pM) and the very slow dissociation rate (kd = 4.5 × 10^−5^ s^−1^), we believe that 2D5 could be a candidate for the generation of scFv-based new CARs. In light of the possibility to target PRAME as an extracellular biomarker, investigating in deeper detail its cellular localization and its interactions with cell-membrane partners would be an interesting future goal. Radioimmunoconjugates and Ab-drug conjugates may also be explored in the context of targeting PRAME-positive cancer cells in an effort to provide effectiveness against cancer cells. Although additional functional studies are required, this new anti-PRAME 2D5 mAbs could be a useful reagent to explore the potential of this class of biotherapeutics in terms of pharmacological efficacy and off-target effects in many PRAME-positive tumors.

## 4. Materials and Methods

The cDNA of PRAME 161–415 and the corresponding pET28a plasmid were generated in the laboratory of Prof. Antonio Leonardi, University of Naples Federico II, Napoli. Reagents and buffers for protein expression and purification were from GE Healthcare (Milan, Italy) and Sigma-Aldrich/Merck-Millipore (Milan, Italy). Reagents for peptide synthesis were from GL Biochem (Shanghai, China), IRIS Biotech GmbH (Marktrewitz, Germany) and Sigma-Aldrich/Merck-Millipore (Milan, Italy). Reagents, sensor chips and the Biacore 3000 instrument for surface plasmon resonance analyses were from GE Healthcare (Milan, Italy). Reagents for ELISA were from Sigma-Aldrich/Merck-Millipore (Milan, Italy). The human osteosarcoma cell line (U2OS) was kindly provided by Prof. Alfredo Budillon (IRCCS-Fondazione Pascale, Naples, Italy). KG1 and L1236 cells were kindly provided by Dr. Antonello Pinto (IRCCS-Fondazione Pascale, Naples, Italy). α-cyano-4-hydroxycinnamic acid (4-CHCA) and tosyl-phenylalanyl-chloromethyl ketone (TPCK)-treated trypsin needed for MALDI-TOF analysis were from Sigma-Aldrich (Milan, Italy). Acetonitrile (Honeywell Riedel-de Haen), trifluoroacetic acid and LC–MS grade water (Honeywell Riedel-de Haen) used for the same purpose were from Fisher Scientific (Milan, Italy). Bio-layer interferometry (BLI) experiments were performed using a BLITZ instrument and ARG2 sensor chips (Alfatest, Milano, Italy). A commercial mouse anti-PRAME antibody was obtained from Abcam (code ab89097, Milan, Italy).

### 4.1. Expression and Purification of Recombinant PRAME Region 161–415

Expression of recombinant human PRAME region 161–415, hereafter *rh*PRAME, was performed in the *E. coli* strain BL21(DE3). Bacterial cells were transformed with the recombinant plasmid pET28a containing the gene corresponding to PRAME region 161–415 (cloning site BamH1/HindIII). The successfully transformed *E. coli* colonies were picked-up and grown at 37 °C in Luria–Bertani (LB) medium supplemented with kanamycin under continuous shaking until the absorbance at 600 nm reached 0.5–0.6. The *rh*PRAME expression conditions were optimized, inducing expression with 1 mM IPTG and incubating the bacteria at 37 °C for 3 h culture to obtain inclusion bodies. *E. coli* cells were harvested by centrifugation at 14,000 rpm for 20 min at 4 °C. The pellet was re-suspended in 50 mM TRIS, 0.15 M NaCl, 1 mM EDTA, 1 mg/mL lysozyme pH 8.0 and kept on the rotor wheel for 30 min at 4 °C, then was further lysed by sonication. The protein was recovered from the inclusion bodies after centrifugation, washing with 20 mM Tris-HCL, pH 8.0, 100 mM NaCl, and final solubilization in a denaturing buffer containing 8 M urea at 4 °C overnight. Insoluble cell debris was again removed by centrifugation. The supernatant fraction was collected and loaded on Ni-NTA agarose resin. The resin was washed with 25 mM TRIS, 4 M urea, 250 mM NaCl, 1 mM DTT, 5 mM Imidazole, pH 7.4. The bound protein was eluted with the same buffer containing 500 mM Imidazole. Fractions containing the purified *rh*PRAME were pooled and dialyzed against the same buffer containing 2 M urea and 1 mM DTT (or 10 mM β mercaptoethanol) overnight at 4 °C. The recovered protein was again dialyzed against 500 mL of PBS buffer containing 1 M urea and 1 mM DTT (or 10 mM β mercaptoethanol) at 4 °C for 4 h and then against the same buffer, but containing only 0.5 M urea overnight at 4 °C since urea removal leads to abundant precipitation. Some protein was recovered and characterized by gel filtration (GF) to assess that it was not aggregated and used to perform immunization of the animals for producing the monoclonal antibodies, the ELISA and the SPR assays. Analytical GF separations were performed on a BIOSEP S-2000 30 × 7.8 mm ID column (Phenomenex, Castel Maggiore, Italy) using 25 mM phosphate buffer pH 7.5 containing 150 mM NaCl as running buffer. The flow rate was 1.0 mL/min; detection was performed at 280 nm. The peak collected from the column was analyzed by 15% SDS–PAGE and dot-blot using an anti-HIs antibody at 5.0 µg/mL. Protein purity was evaluated during the purification by 15% SDS–PAGE analysis staining with Coomassie brilliant blue.

### 4.2. Immunization of Mice and Generation and Purification of mAbs against rhPRAME

BALB/c mice were housed and handled according to the institutional guidelines (Project identification code 2013/0038120, approved by the Ethical Animal Care and Use Committee, University of Naples “Federico II”. Date of approval 24 April 2013). Four 5-week old BALB/c mice (Jackson Lab) were immunized with 100 μg of highly purified *rh*PRAME, from which the urea was removed following extensive dialysis and recovery of the residual soluble protein. The protein was emulsified with Complete Freund’s adjuvant. To obtain hybridomas secreting anti-PRAME antibodies, we operated as previously described [27,28,29,30]. Hybridoma supernatants were screened by ELISA for binding to *rh*PRAME, and those secreting antibodies with strong reactivity were re-cloned twice by limiting dilution and tested in dose-dependent ELISAs to confirm binding. Subcloned hybridomas were cultured in OPTI-MEM medium containing 10% FBS and adapted gradually to serum-free cell medium. The selected hybridoma was transferred to bioreactors (INTEGRA Biosciences AG, CH-7000 Chur, Switzerland) for large-scale antibody production. Monoclonal antibodies were purified by protein G affinity and size exclusion chromatography (not shown), as reported previously [27,28,29,30]. The mAb concentration was determined by Nanodrop 200 °C, and the purity and homogeneity were detected by 15% SDS–PAGE.

### 4.3. ELISA Binding Assays of 2D5 mAb to rhPRAME and to Biotin-PRAME Peptides

ELISA binding assays were performed as described previously [27,28,29,30,31]. For dose–response binding assays, *rh*PRAME was coated at three concentrations (17.4 nM, 35 nM and 174 nM) on microtiter ELISA plates in triplicate wells. The selected purified anti-PRAME 2D5 mAb was tested at increasing concentrations between 0.17 nM and 13.3 nM. An HRP-conjugated anti-mouse antibody (Bio-Rad, Milano, Italy) was used as the secondary antibody to detect the bound mAb. OPD was used as a chromogenic substrate. Comparative binding experiments between mAb 2D5 and a commercial mouse anti-PRAME antibody (code ab89097, Abcam, Milano, Italy) were performed coating the protein at 3 concentrations (0.5, 1.0 and 5.0 µg/mL) and using the two antibodies at 0.5 µg/mL. Detection of bound antibodies was performed using an HRP-conjugated anti-mouse antibody (Bio-Rad, Milano, Italy) at 1.0 µg/mL. For ELISA epitope binding experiment, the 2D5 mAb was coated at 6.7 nM and dose–response binding was tested using the N-terminally modified peptide PRAME (202–212), named biotin-PRAME (202–212) (See Table S1), at concentrations ranging between 46 nM and 6.14 µM. Streptavidin-HRP (Sigma-Aldrich, Milano, Italy) was used at the final concentration of 0.15 µg/mL diluted in PBS. Detection was achieved using an OPD tablet (Sigma-Aldrich, Milano, Italy), the peroxidase reaction was stopped with 50 µL/well of 2.5 M H_2_SO_4_, and the optical density was measured at 490 nm, using a multiwell plate reader (BioTek, Winooski, VT, USA). All experiments were performed 3 times and in triplicate. The binding of the mutated biotin-PRAME peptides (reported in Table S1) was evaluated in the same conditions. Data were fitted using GraphPad ver. 6.0 by applying a nonlinear regression analysis algorithm.

### 4.4. SPR-Based Affinity Measurements

All SPR analyses were performed on a Biacore 3000 instrument (GE Healthcare), using CM5 sensor chips with immobilized a highly purified (no urea was present) fraction of the recombinant protein. HBS-EP (10 mM HEPES, pH 7.4, 150 mM NaCl, 3 mM EDTA, 0.005% surfactant P20) was used as running buffer. The immobilization was carried out following the canonical amino coupling chemistry [32], operating at a flow rate of 5 µL/min, using the Wizard application. The chip surface was activated with a 50:50 (*v/v*) EDC/NHS mixture (containing 0.4 M EDC and 0.1 M NHS). *rh*PRAME was immobilized at 10 µg/mL in 10 mM NaAc, at pH 4.5. The remaining active ester groups were finally blocked with 1 M ethanolamine-HCl at pH 8.5. A reference channel was opportunely prepared to perform the same procedure without the ligand. The binding ability of the selected anti-PRAME 2D5 mAb was tested at increasing concentrations ranging between 0.25 nM and 2.0 nM. All analyses were carried at 25 °C, at a 20 µL/min constant flow rate, and injecting 60 µL volume of analyte solutions opportunely diluted at different concentrations. HBS-EP was used to prepare the antibody samples. NaOH at 5 mM was used to regenerate the chip surface after the binding with the antibody. For each individual injection, experimental sensorgrams were aligned, subtracted of blank signal and were then overlaid. All mathematical manipulations and fitting operations were performed using the BIA evaluation software, version 4.1 (GE Healthcare) and assuming a 1:1 Langmuir binding model. The final apparent K_D_ was determined by calculating the average of the values obtained in separate experiments at 0.5 nM, 1.0 nM, 1.5 nM and 2.0 nM where the kd (koff) and ka (kon) were not grossly different.

### 4.5. Western Blot Analyses of Endogenous PRAME in Extracts of Cell Expressing and not Expressing the Protein

Western blotting analyses were performed using standard procedures. Cells were grown as previously reported [33,34,35]. Cell extracts from KG1 (PRAME-negative cells) and L1236 cells (PRAME-positive) were prepared as previously described [28,35]. Proteins were separated on a 15% SDS–PAGE gel and transferred to a polyvinylidene difluoride membrane (Millipore, USA). After blocking with 5% NFDM, the membrane was incubated with the anti-PRAME 2D5 mAb at 1.0, 2.0 and 5.0 µg/mL overnight at 4 °C. Similarly, Western blotting analyses were performed on U20S cells (PRAME-positive using *rh*PRAME as a positive control. Hybridization was set up using the 2D5 mAb at 5.0 µg/mL. Detection was performed using an ECL substrate kit (Pierce, Thermo Fisher, Waltham, MA, USA) according to the manufacturing procedures. Images were acquired using the ChemiDoc imaging system (Bio-Rad).

### 4.6. Flow Cytometry Analysis

The human osteosarcoma cell line (U2OS) was grown in DMEM supplemented with 10% fetal bovine serum (FBS), 2 mM L-glutamine, 100 U/mL penicillin, 100 µg/mL streptomycin (EuroClone, Milano, Italy) and was maintained in humidified air containing 5% CO_2_ at 37 °C. Adherent cells at about 70% confluence were detached using 0.25% trypsin, 2 mM EDTA (EuroClone, Milano, Italy), centrifuged and suspended in PBS1x. For surface receptor labeling, cell aliquots (5 × 10^5^ cells) were treated in the same manner with the commercial mouse anti-PRAME antibody (code ab89097, Abcam, Milano, Italy), the anti-PRAME 2D5 mAb or a mouse IgG isotype control antibody (Cell signaling Technologies, Boston, MA, USA), at the different concentrations. For intracellular staining, cells were fixed and permeabilized using an intrastain kit (Dako, Glostrup, Denmark), according to the manufacturer’s instructions and incubated again with the primary antibodies. After washing, cells were treated with a FITC-conjugated secondary antibody (Jackson ImmunoResearch Laboratories Inc, Baltimore, USA) and diluted according to the manufacturer’s recommendations. Labeled cells were washed and analyzed using a flow cytometer equipped with a 488 nm argon laser (FACScan, Becton Dickinson, CA, USA). A total of 20,000 events for the sample were collected, and values of fluorescence intensity were obtained from the histogram statistic of CellQuest software. All FACS analyses were performed at least 3 times.

### 4.7. Bio Layer Interferometry-Based Epitope Capture Assay

**Scheme 1:**
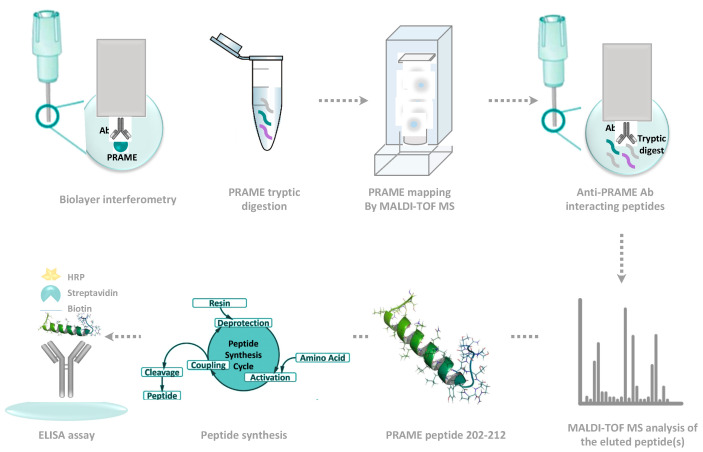
Schematic representation of the approach followed to identify the epitope of mAb 2D5.

The anti-PRAME 2D5 mAb was immobilized on an ARG2 BLI sensor tips as previously reported following the EDC/NHS method [36]. The antibody was diluted at a concentration of 5.0 µg/mL in sodium acetate buffer 10 mM, pH 5.0 (4.0 µL) and exposed to the preactivated sensor chip for 3 min. The surface was deactivated with ethanolamine 0.5 M pH 8.0 and extensively washed with PBS1x buffer (4.0 µL). One single dose experiment was carried out to assess the binding of the protein on the new sensor chip in the presence of urea. The binding of *rh*PRAME was tested at 1.0 µM, the concentration of the digested protein used in the capture experiment. Capture experiments were performed using the fragment mixture obtained following digestion with trypsin (see below) at the final concentration of 1.0 µM. The experiment was repeated 10 times, accumulating the eluted fraction in a single pooled fraction. For each run, the tryptic peptide mixture (4.0 µL) was exposed to the Anti-PRAME 2D5 mAb derivatized sensor chip for 3 min. After washing the chip with PBS for 1 min, the captured peptides were eluted with 4 µL of 0.1% TFA in H_2_O. The pooled fraction containing the eluted peptides was lyophilized and reconstituted in 30 µL of water/acetonitrile (70:30, *v/v*, containing 0.1% TFA) and analyzed by MALDI-TOF MS. See Scheme 1.

The binding of the biotinylated PRAME peptides (See Table S1) to the 2D5 antibody was similarly performed using SA BLI sensor chips. Peptides were immobilized at 20 µg/mL in PBS running buffer by 3 min exposure (contact time) of the solution to the sensor tips. Bound nonspecific material was washed by short washing (10 sec pulses) with NaOH 5 mM. The chips were extensively equilibrated with PBS. An SA sensor chip functionalized with biocytin was used as a negative control to test the monoclonal antibody specificity. Dose–response binding experiments with the soluble mAb were performed in the concentration range between 0.5 and 7.5 µM (volume 4 µL) in PBS buffer. Each individual assay was completed performing the following steps: (i) exposure to running buffer to acquire the initial baseline (baseline, exposure time 30 s); (ii) exposure to antibody solutions (association, volume 4.0 µL, exposure time 120 s); (iii) exposure to running buffer (dissociation, exposure time 120 s); (iv) exposure to 5 mM NaOH (regeneration, exposure time 10 s). The shaker speed was set to 2000 rpm according to the manufacturer’s instructions. Data were exported from the BLItz Pro 1.2 software and re-plotted with GraphPad Prism, version 5.00, GraphPad software (San Diego, CA, USA). Plateau values of binding as reflected by changes in optical thickness (nm) at 140 s were used to calculate the affinity constant (KD) by applying a nonlinear curve fitting and one binding site hyperbola as a model (GraphPad Prism).

### 4.8. Tryptic Digestion and MS Analysis

*rh*PRAME (5.0 µg) in around 150 µL of 25 mM Tris-HCl buffer, pH 8.2 (around 1.0 µM), containing 0.5 M urea, 40 mM NaCl and 0.3 mM DTT was further added of DTT up to 10 mM and left for 1 h at 55 °C. The protein was next alkylated with 7.5 mM IAM (iodoacetamide) by incubating the solution for 30 min at room temperature in the dark. The enzymatic hydrolysis was performed by adding TPCK-treated trypsin (4.0 ng/µL) at an enzyme/substrate (E/S) ratio of 1:50 (*w/w*) and by incubating the sample at 37 °C for 16 h. The peptide mixture was analyzed by matrix-assisted laser desorption ionization time-of-flight (MALDI-TOF) mass spectrometry to identify the protein fragment generated, as previously reported [37]. Briefly, 1.0 μL of samples were mixed with 1.0 μL of saturated α-cyano-4-hydroxycinnamic acid matrix solution at 10 mg/mL in acetonitrile/water (1:1, *v/v*), containing 0.1% TFA. A droplet of the resulting mixture (1.0 μL) was placed on the MALDI-TOF micro MX (Waters, Manchester, UK) target plate and dried at room temperature. Once the liquid was completely evaporated, samples were loaded into the mass spectrometer and analyzed. In reflectron mode, the instrument was externally calibrated using a tryptic alcohol dehydrogenase digest (Waters, Milan, Italy). For linear mode analysis, a 4-point external calibration was applied using an appropriate mixture (10 pmol/μL) of ProteoMass ACTH Fragment, insulin, cytochrome C and horse Mb as calibration standard (Sigma-Aldrich, Milan, Italy). All spectra were processed and analyzed by using the Mass Lynx 4.1 software. The tryptic peptides captured by the mAb 2D5 and eluted from the sensor chip were similarly analyzed.

### 4.9. Peptide Synthesis

The synthetic biotin-PRAME (202–212) peptide (Bio-βAla-KKNVLRLCCKK, Table S1) and the mutated variants (See Table S1), amidated at the C-terminus, were prepared by the Fmoc/tBu methodology as reported elsewhere [38,39] and purified by RP-HPLC. Identity was assessed by LC–MS analyses. The peptides were biotinylated on the N-terminus using biotin-N-hydroxysuccinimide (biotin-NHS, Sigma-Aldrich, Milan, Italy) dissolved in dimethylformamide (DMF) containing 5% di-iso-propyl-ethylamine [39]. After assembly, the peptides were cleaved from the Rink-amide resin and purified by RP-HPLC, as previously reported [36]. The final products were identified by ESI-TOF LC–MS analyses using an Agilent 1290 Infinity LC System coupled to an Agilent 6230 TOF mass spectrometer. The experimental MWs were in agreement with the theoretical values (See Table S1).

## 5. Conclusions

We generated a new anti-PRAME monoclonal antibody by immunizing mice with a recombinant fragment of the human protein encompassing residues 161–415. One high-affinity antibody named 2D5 (K_D_ = 35 pM) was isolated and used in a number of assays to detect the full-length protein by cytofluorimetry on the surface of cell membranes and by Western blotting in cell extracts. Using an approach based on the immobilization of the antibody on BLI sensor chips and immunoaffinity capture of PRAME peptides obtained after trypsin digestion, we also identified a unique protein fragment (region 202–212) that is able to bind the antibody. By using this fast, inexpensive and novel method, the protein region involved in antibody recognition was, therefore, rapidly delineated, also taking advantage of the small volume (4 µL) of the sensor cuvette and of the lack of fluidics of the label-free system, which enables accumulation of the eluted material following repeated binding-elution cycles. Given the high affinity and the ability to recognize the protein on the surface of cell membranes, the antibody has the potential to become a good template for developing T cell-based immunotherapies.

## Figures and Tables

**Table 1:** SPR kinetic rates and apparent affinity binding constants of anti-PRAME mAb 2D5 for *rh*PRAME.

nM	Ka (1/Ms)	Kd (1/s)	K_D_ (M)	SE (RI)
0.25	3.53 × 10^5^	2.11 × 10^−4^	5.97 × 10^−10^	0.153
0.5	2.77 × 10^6^	4.36 × 10^−5^	1.57 × 10^−11^	0.056
0.7	3.60 × 10^5^	9.73 × 10^−6^	2.71 × 10^−11^	0.049
1	2.92 × 10^6^	3.09 × 10^−5^	1.06 × 10^−11^	0.041
1.5	1.18 × 10^6^	4.16 × 10^−5^	3.51 × 10^−11^	0.028
2	8.21 × 10^5^	6.43 × 10^−5^	7.83 × 10^−11^	0.048
average			3.49 × 10^−11^ *	

* The average was calculated excluding the deviating kd values obtained at 0.25 nM and 0.7 nM.

## Data Availability

Not applicable.

## Supplementary Materials

Supplementary materials can be found at https://www.mdpi.com/1422-0067/22/6/3166/s1.

## Abbreviations

PRAME	Preferentially expressed antigen in melanoma
EMT	Epithelial-to-mesenchymal transition
TCR	T cell receptor
FACS	Fluorescence assisted cell-sorting
SPR	Surface plasmon resonance
ELISA	Enzyme-linked immunosorbent assays
K_D_	Thermodynamic dissociation constant
K_d_	Kinetic dissociation constant
MALDI-TOF	Matrix Assisted Laser Desorption Ionization Time of Flight
