# Supplementary material for: Development of a New Highly Selective Monoclonal Antibody against Preferentially Expressed Antigen in Melanoma (PRAME) and Identification of the Target Epitope by Bio-Layer Interferometry
Source: Int J Mol Sci. 2021 Mar 20;22(6):3166. doi: 10.3390/ijms22063166 (PMC8003813; doi:10.3390/ijms22063166)
Supplement: Supplementary file 1 [file ijms-22-03166-s001.pdf]

Supplementary Information

Development of a New Highly Selective Monoclonal Antibody against Preferentially Expressed Antigen in Melanoma (PRAME) and Identification of the Target Epitope by Bio-Layer Interferometry

Jwala Priyadarsini Sivaccumar [1], Antonio Leonardi [2], Emanuela Iaccarino [1], Giusy Corvino [1], Luca Sanguigno [2], Angela Chambery [3], Rosita Russo [3], Mariangela Valletta [3], Debora Latino [1], Domenica Capasso [4], Nunzianna Doti [1], Menotti Ruvo [1,*] and Annamaria Sandomenico [1,*]

1 Istituto di Biostrutture e Bioimmagini, CNR, 80134 Napoli, Italy; jwala.priyadarsini@gmail.com (J.P.S.); emanuela.iaccarino@gmail.com (E.I.); giusycorvino1986@gmail.com (G.C.); latinodebora@gmail.com (D.L.); nunzianna.doti@cnr.it (N.D.)
2 Dipartimento di Medicina Molecolare e Biotecnologie Mediche, Università degli Studi di Napoli Federico II, 80142 Napoli, Italy; leonardi@unina.it (A.L.); lucasanguigno@libero.it (L.S.)
3 Dipartimento di Scienze e Tecnologie Ambientali, Biologiche e Farmaceutiche (DISTABIF), Università L. Vanvitelli, 80100 Caserta, Italy; angela.chambery@unicampania.it (A.C.); rosita.russo@unicampania.it (R.R.); mariangela.valletta@unicampania.it (M.V.)
4 Centro di servizio di Ateneo per le Scienze e Tecnologie per la Vita (CESTEV), Università di Napoli Federico II, 80145, Napoli, Italy; domenica.capasso@unina.it
* Correspondence: menotti.ruvo@unina.it (M.R.); annamaria.sandomenico@cnr.it (A.S.)

INDEX

Figure S1
Figure S2
Figure S3
Figure S4
Figure S5
Figure S6
Figure S7
Figure S8

MGSSHHHHHHSSGLVPRGSHMASMTGGQQMGRGSEF^{161}VDGLSTEAEQPFIPVEVLV
DLFLKEGACDELFSYLIEKVKR^{202}**KKNVLRLCCKK**^{212}LKIFAMPMQDIKMILKMVQLDSIEDL
EVTCTWKLPTAKFSPYLGQMINLRRLLLSHIHASSYISPEKEEQYIAQFTSQFLSLQCLQALYV
DSLFFLRGRLDQLLRHVMNPLETLSITN**C**RLSEGDVMHLSQSPSVSQLSVLSLSGVMLTDVS
EPQ ALLERASATLQDLVFDECGITDDQLLALLPSLSHCSQLTT LSFYG^{415}

Figure S1. Amino acidic sequence of rhPRAME region 161-415. In bold is evidenced the N-terminal histidine Tag. In red is reported the identified epitope region.

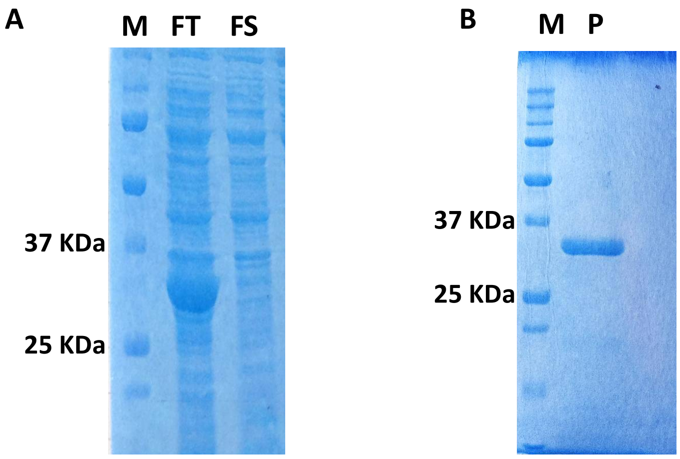

Figure S2. 12% SDS PAGE analysis under reducing conditions of the total E. coli extract following the expression of human PRAME (A). 15% SDS PAGE analysis under reducing conditions of the fraction recovered after affinity purification (B) of recombinant protein. In A. Lane M: Precision Plus Protein marker (250-10 kDa, Bio-Rad). Lane FT: total fraction. Lane FS: soluble fraction. In B. Lane M: Precision Plus Protein marker (250-10 KDa; Bio-Rad) and lane P, purified protein under reducing conditions.

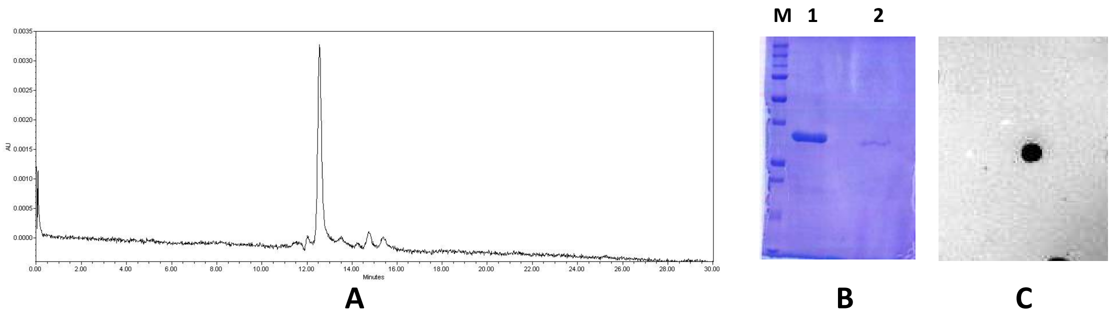

Figure S3. A. Size exclusion profile of rhPrame in native running buffer (25 mM, phosphate, 150mM NaCl, pH=7.5; **B**. 15% SDS-PAGE analysis of the protein collected from the GF separation: M, marker 15-150 kDa; rhPRAME inject (1) and sample collected from the GF and analysed after lyophilization (2); **C**. Dot blot analysis of the protein sample collected from the GF analytical run as in Figure A and lyophilized. Detection was performed with an anti-His antibody.

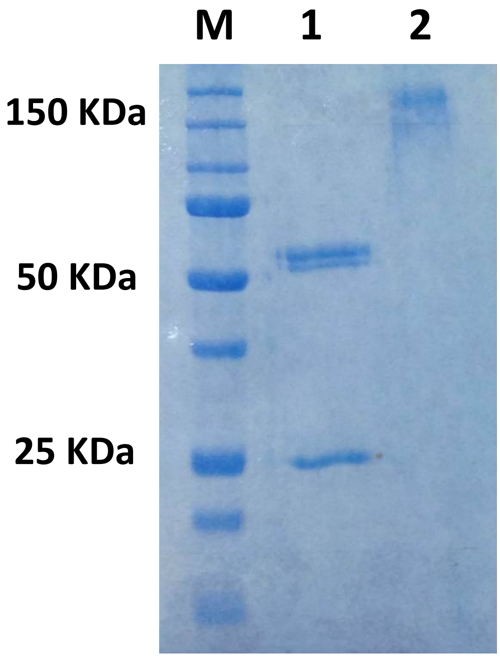

Figure S4. 15% SDS PAGE analysis of the 2D5 anti-PRAME purified monoclonal antibody under reducing (lane 1) and non-reducing conditions (lane 2). Lane M: Precision Plus Protein marker (250-10 kDa, Bio-Rad).

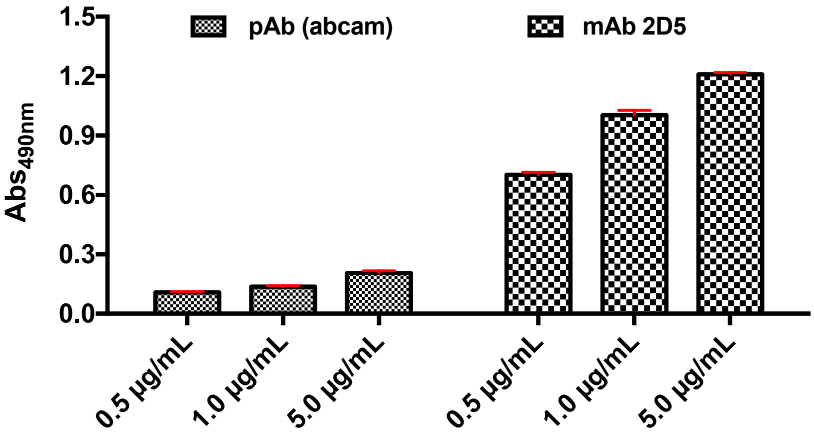

Figure S5. Comparative ELISA binding assays to rhPRAME performed using mAb 2D and a commercial anti-PRAME polyclonal antibody (Abcam, code ab89097). Conditions are those reported in the section of Methods.

Peptide Name	Peptide sequence	M.W. theor. (amu)	M.W. exp. . (amu)
Biotin-PRAME [202-212]	Bio-βAla-KKNVLRLCCKK	1627.31	1627.83
Mutant K203A-R207A-K211A	Bio-βAla-K**A**NVL**A**LCC**A**K	1428.96	1429.66
Mutant V205A-L206A-L208A	Bio-βAla-KKN**AA**R**A**CCKK	1516.01	1516.71
Mutant C209S-C210S	Bio-βAla-KKNVLRL**SS**KK	1596.18	1597.00

Table S1. Biotinylated-PRAME peptides. Peptides are reported with single letter codes. Bio stands for biotin and βAla represents a βAlanine residue. Mutated residues are reported in bold red.

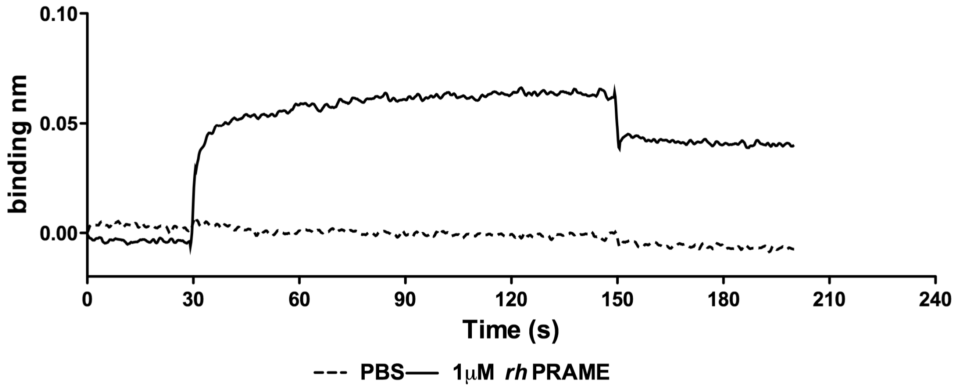

Figure S6. BLI measurements showing the binding of rhPRAME at 1.0 μM to immobilized anti-PRAME 2D5 mAb.

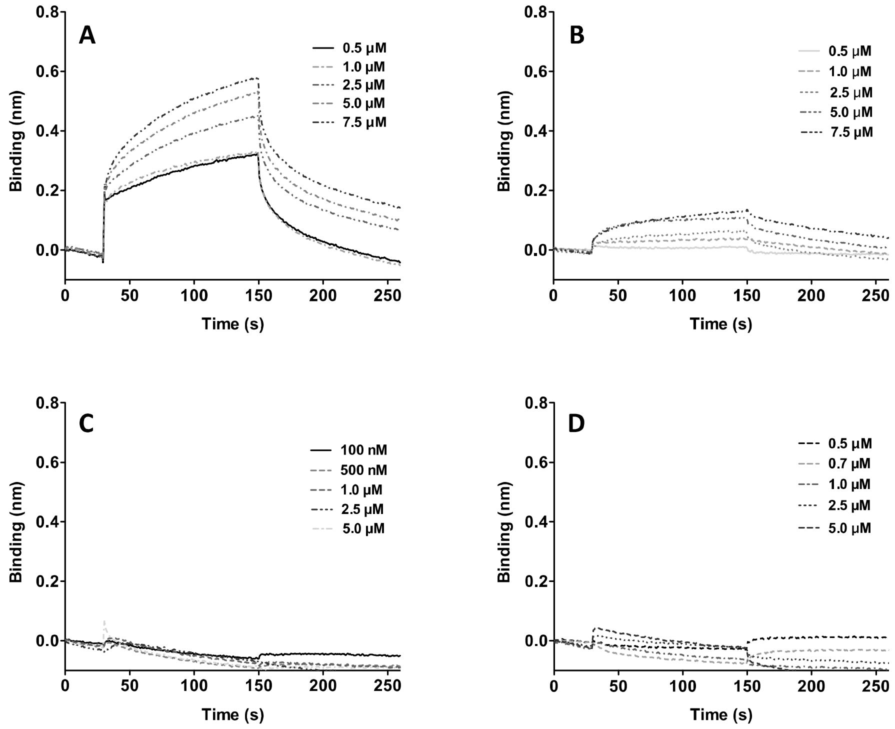

Figure S7. BLI measurements of the anti-PRAME 2D5 mAb to the biotinylated PRAME peptides immobilized on SA BLI sensor chips. **A**. Binding of 2D5 at 0.5 μM, 1.0 μM, 2.5 μM, 5.0 μM and 7.5 μM to biotin-PRAME[202-212]; **B**. Binding of 2D5 at 0.5 μM, 1.0 μM, 2.5 μM, 5.0 μM and 7.5 μM to biotin-PRAME[202-212]-K203A-R207A-K211A; **C**. Binding of 2D5 at 0.1 μM, 0.5 μM, 1.0 μM, 2.5 μM and 5.0 μM to biotin-PRAME[202-212]-V205A-L206A-L208A; **D**. Binding of 2D5 at 0.5 μM, 0.7 μM, 1.0 μM, 2.5 μM and 5.0 μM to biotin-PRAME[202-212]-C209S-C210S.

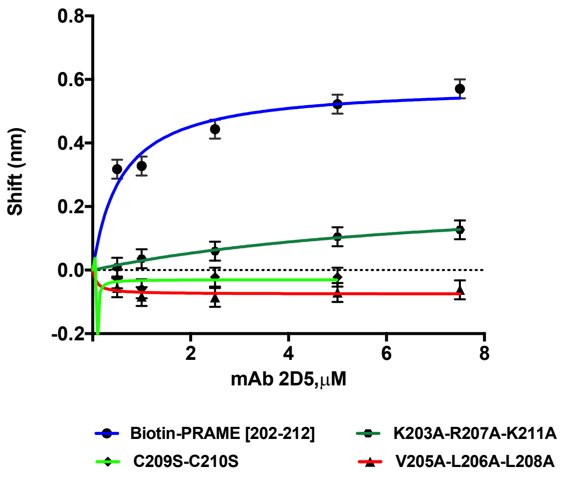

Figure S8. Plateau values of binding as reflected by changes in optical thickness (nm) at 140 s plotted as a function of antibody concentration. Data were used to calculate the affinity constant (K_D) by applying a non-linear curve fitting and one binding site hyperbola as model. The estimated K_D for PRAME [202-212] was 0.59 μM, very similar to that estimated by ELISA (0.55 μM).
